# Integrated analysis of genome-wide gene expression and DNA methylation profiles reveals candidate genes in ovary endometriosis

**DOI:** 10.3389/fendo.2023.1093683

**Published:** 2023-03-23

**Authors:** Lei Lei, Xinxin Xu, Chengchen Gong, Bowen Lin, Fang Li

**Affiliations:** ^1^ Department of Gynecology, Shanghai East Hospital, Tongji University School of Medicine, Shanghai, China; ^2^ Department of Dermatology, Shanghai East Hospital, Tongji University School of Medicine, Shanghai, China; ^3^ Department of Cardiology, Shanghai East Hospital, Tongji University School of Medicine, Shanghai, China; ^4^ Research Center for Translational Medicine, East Hospital, Tongji University School of Medicine, Shanghai, China

**Keywords:** DNA methylation, gene expression, epigenetics, endometriosis, multi-omic analysis

## Abstract

**Background:**

The incidence of endometriosis (EMs), a common disease in gynecology, has increased over the years. Women suffer from the symptoms caused by EMs, such as chronic pelvic pain, dysmenorrhea, and infertility. However, the etiology and pathophysiology of EMs remain unclear. This study aimed to identify candidate genes of endometriosis through integrated analysis of genome-wide gene expression and DNA methylation profiles.

**Results:**

Eutopic and ectopic endometrial tissues were collected from patients who were diagnosed as ovarian EMs. Genome-wide methylation profiling identified 17551 differentially methylated loci, with 9777 hypermethylated and 7774 hypomethylated loci. Differentially methylated loci were mainly concentrated in the gene body and intergenic regions. Genome-wide gene expression profiling identified 1837 differentially expressed genes (DEGs), with 1079 genes upregulated and 758 downregulated in ectopic groups. Integrated analysis revealed that DNA methylation was negatively correlated to gene expression in most genomic regions, such as exon, 3’UTR, 5’UTR, and promoter. We also identified promoter-related (53 downregulated and 113 upregulated) and enhancer-related DMGs (212 downregulated and 232 upregulated), which were significantly correlated to the gene expression. Further validation of the top-ranked genes belonging to differentially methylated genes (DMGs) and DEGs revealed that *TMEM184A*, *GREM2*, *SFN*, *KIR3DX1*, *HPGD*, *ESR1*, *BST2*, *PIK3CG* and *RNASE1* were significant candidate genes in ovarian endometriosis.

**Conclusion:**

Our study revealed the significance of DNA methylation in the gene expression in ovary endometriosis, which provides new insights and a molecular foundation for understanding the underlying mechanisms of endometriosis.

## Introduction

Endometrial tissue that exhibits growing activity outside the uterine cavity and grows abnormally is defined as endometriosis (EMs) (1). Lesions typically occur in the peritoneal cavity, including the ovaries, uterorectal depressions, and uterosacral ligaments (2). EMs affected 5% to 15% of women of reproductive age (3). Patients with EMs suffer a series of symptoms, such as pelvic pain, dysmenorrhea, and infertility (4). EMs exhibit biological behaviors of malignant tumors as a benign disease, such as cell proliferation, implant growth, infiltration, distant metastasis, and recurrence (2). The current mainstream etiological theories cannot fully explain the pathogenesis of EMs, including ‘menstrual blood reflux theory’, ‘lymphatic and venous dissemination theory’ (5), and ‘body cavity epithelial metaplasia theory’ (6), immunological mechanism, angiogenesis mechanism, cell proliferation, and apoptosis imbalance mechanism, and in-place endometrial determinism (7). In recent years, the incidence of EMs has significantly increased, and the patient’s onset age tends toward younger, calling for a deeper understanding of the pathogenesis of EMs.

DNA methylation is important in the regulation of gene expression (8). Several studies reported that DNA methylation might play an essential role in the pathogenesis of EMs (9). With the development of sequencing techniques, genome-wide methylation differences between eutopic and ectopic endometrial tissues have been investigated in recent years (10–14). However, few studies linked genome-wide DNA methylation with the changes in gene expression in EMs (10, 15), suggesting DNA methylation changes were associated with altered gene expression in endometrial function or dysfunction. Herein, we integrated the genome-wide DNA methylation with the gene expression of EMs to find candidate genes in EMs.

## Materials and methods

### Patient enrollment and sample collection

Thirteen female patients who were diagnosed as ovarian EMs were enrolled. DNA methylation profiles were performed on 6 patient specimens, RNA sequencing was performed on 3 patient specimens, and quantitative reverse transcription PCR (qRT-PCR) verifications were performed on 7 patient specimens. Patients (Hans, 27-42 years old) in Shanghai East Hospital undergoing laparoscopic and postoperative pathological diagnosis of ovarian EMs were selected. The typical ectopic endometrial tissue and the matched eutopic endometrium from each patient were collected in the proliferation phase. All the participants had regular menstrual cycles and had not received hormonal medication 3 months before surgery. The participants had no history of other organic diseases, such as hypertension, diabetes, tumors, chronic infections, etc. Patients with benign gynecologic diseases (leiomyoma, ovarian serous cyst, PCOS) were excluded. All patients provided written informed consent in this study. This study was approved by the Ethics Committee of the Shanghai East Hospital of Tongji University.

### DNA and RNA extraction

All the samples were kept in RNAlater (Ambion, AM7020) when obtained during laparoscopic surgery. Ectopic and eutopic tissues were extracted for genomic DNA detection according to the manufacturer’s instructions (QIAamp DNA Mini Kit, 51304). Total RNA was extracted from ectopic and eutopic tissues using RNAiso plus reagent (Takara, 9109) following the manufacturer’s instructions. Briefly, tissues were grinded and crushed in liquid nitrogen, and then added 300 µL chloroform/isoamyl alcohol (24:1) and mixed thoroughly. After centrifuging, the supernatant was moved to a 1.5 mL centrifuge tube, mixed with isopropyl alcohol, and then purified to RNA for the subsequent experiments.

### DNA methylation array

At least 500 ng genomic DNA was extracted and bisulfate converted (Zymo EZ-96 DNA Methylation-Direct Kit, D5023) before being processed to the methylation array. Illumina Human Methylation 450 K BeadChips were applied to get the whole genome methylation states of the samples. The experimental data were sorted out and encoded, and the data was double-converted and double-calibrated by EpidData. Background adjustment and quantile normalization pretreatment were performed according to the raw data of each probe methylation site of the Illumina methylated HM450K BeadChip chip. Then the degree of methylation at the probe level was analyzed. β value was calculated and normalized to quantify the degree of methylation. In this study, the average β value ≥ 0.2 and *P*-value < 0.05 was considered as significant.

### Differential methylation analysis

After standardizing the original methylation data, the Pearson Correlation Coefficiency (PCC) was used to evaluate the methylation profile correlation between the ectopic and eutopic groups. The correlation between ectopic and eutopic samples was high, indicating the overall reproducibility of the samples. Since not all the samples were in accordance with normal distribution, the Wilcox test was applied to do differential methylation analysis. The criteria for screening the difference site need to meet both (1) the original *P* < 0.05 and (2) the difference of ectopic and eutopic group beta value (delta beta value) greater than or equal to 0.2. The results were also calculated with FDR (false discovery rate) corrected by multiple hypothesis testing to assess differential methylation levels. The differential DMR biological function analysis was performed using the hypergeometric distribution algorithm or Fisher test. Biological pathway analysis was enriched in KEGG (Kyoto Encyclopedia of Genes and Genomes, www.genome.jp/kegg/) database.

### RNA-sequencing and analysis

RNA libraries were constructed with a VAHTS Universal V6 RNA-seq Library Prep Kit for MGI (Vazyme, NRM604). DNBSEQ-T7 high-throughput sequencing platform of BGI was used for sequencing. The raw data was filtered to clean data after trimming low quality reads and adapters. Clean data were mapped to mm10 mouse reference genome using Hisat2 software (version 2.2.1). The reads were quantified by featureCounts (version 2.0.1). The differential gene analysis was performed by Deseq2 (version 1.32.0). Genes with adjusted *P* value < 0.05 and the abs (Log2(Fold Change)) ≥ 1 was considered as differentially expressed genes (DEGs). The enrichment of Gene Ontology (GO) was conducted by clusterProfiler package (v4.0.5).

### Quantitative reverse transcription PCR

Total RNA was extracted from ectopic and eutopic samples. cDNA was obtained after reverse transcription reversal by Evo M-MLV RT Kit (Accurate Biology Co. Ltd, AG11601). The qRT-PCR was performed by Evo M-MLV One Step RT-PCR Kit (Accurate Biology Co. Ltd, AG11607). Each target gene was compared to β-actin. The expression of target mRNA was calculated based on 2^-ΔΔCt^ method. The primers used in this study were listed in the **Supplementary Table S2**.

### Statistical analysis

All the statistical analyses were performed using R software (version 4.0.1). *P*-values less than 0.05 were considered statistically significant. The Kolmogorov-Smirnov test was applied to evaluate the normality of the distribution of the variables. For qRT-PCR, statistical analyses were conducted using a student *t*-test (data with normal distribution) or Mann-Whitney test (data with skewed distribution) as appropriate by GraphPad Prism software 8.0 (GraphPad Software Inc).

## Results

### Genome-wide DNA methylation changes between eutopic and ectopic endometrial tissues

**Figure 1:**
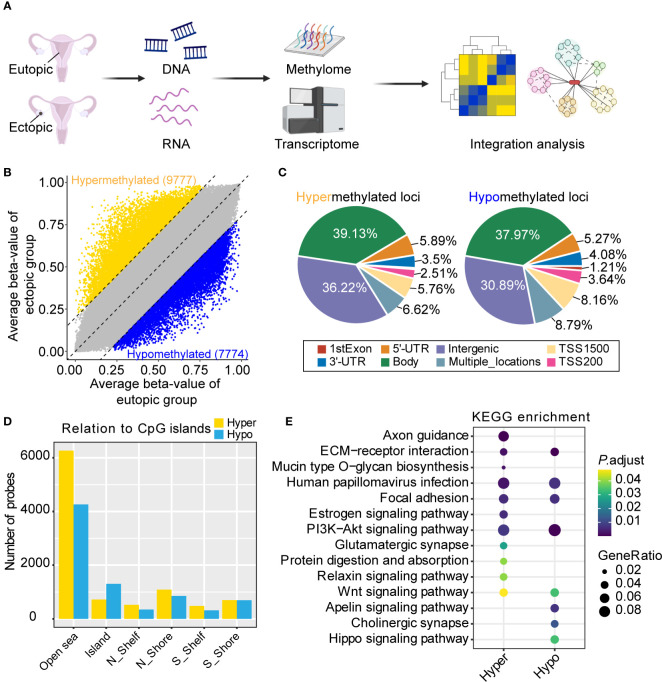
Genome-wide DNA methylation analysis of eutopic and ectopic endometrial tissues. **(A)** Schematic diagram of the study. Created with BioRender.com. **(B)** Differential methylated loci in eutopic and ectopic endometrial tissues. **(C)** The distribution of hypermethylated (left) and hypomethylated (right) loci in ectopic endometrial tissues. **(D)** CpG island probe distribution of hypermethylated and hypomethylated loci in ectopic endometrial tissues. **(E)** Kyoto Encyclopedia of Genes and Genomes (KEGG) pathway analysis of the hypermethylated and hypomethylated loci in the ectopic group.

We first obtained the genome-wide DNA methylation changes of eutopic and ectopic endometrial tissues (**Figure 1A**). The clinical characteristics of the patients were summarized in **Supplementary Table S1**. A total of 17551 differentially methylated loci were identified, of which 9777 were hypermethylated and 7774 were hypomethylated (**Figure 1B**). To get view of the distribution of differentially methylation loci, we annotated the locations to different functional elements in the genome. Hypermethylated and hypomethylated loci in ectopic groups were mainly located in gene body (39.13% of hypermethylated loci, and 37.97% of hypomethylated loci) and intergenic regions (36.22% of hypermethylated loci, and 30.89% of hypomethylated loci) (**Figure 1C**). Furthermore, the CpG island probe distribution of both hypermethylated and hypomethylated loci occurred in the OpenSea region (**Figure 1D**). Next, we performed KEGG (Kyoto Encyclopedia of Genes and Genomes) pathway analysis of differentially methylated loci. Both hypermethylated and hypomethylated genes in ectopic were enriched in ECM-receptor interaction, human papillomavirus (HPV) infection, focal adhesion, PI3K-Akt signaling pathway and Wnt signaling pathway. Moreover, axon guidance, estrogen signaling pathway, glutamatergic synapse, protein digestion and absorption, and relaxin signaling pathway were only enriched in hypermethylated genes. Besides, apelin signaling pathway, cholinergic synapse and Hippo signaling pathway were only enriched in hypomethylated genes in the ectopic group (**Figure 1E**). The above results indicated that DNA methylation was associated with various pathways in the eutopic and ectopic endometrial tissues.

### Transcriptome analysis of ectopic and eutopic samples

**Figure 2:**
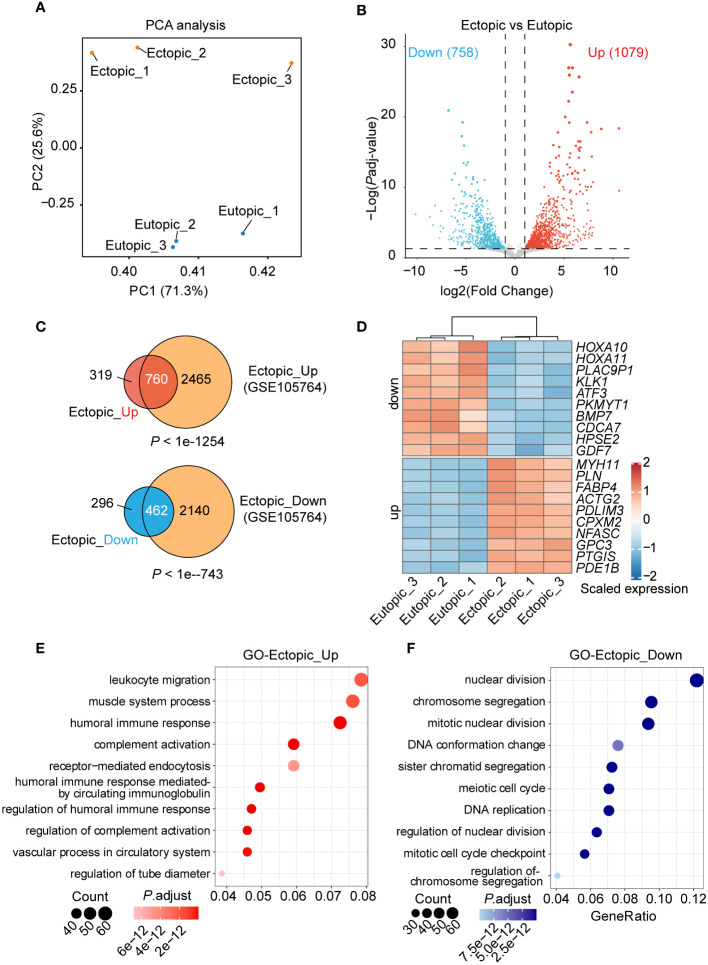
Genome-wide gene expression analysis of eutopic and ectopic endometrial tissues. **(A)** Principal components analysis (PCA) analysis of the eutopic and ectopic samples. **(B)** Differentially expressed genes (DEGs) analysis of eutopic and ectopic samples. **(C)** Venn plots of the DEGs and DEGs in the GSE105764 dataset. **(D)** Heatmap of the top 10 upregulated and downregulated DEGs in RNA-seq. **(E)** GO analysis of the upregulated genes in ectopic samples. **(F)** GO analysis of the downregulated genes in ectopic samples.

To view the transcriptional difference between ectopic and eutopic endometrial tissues. We performed RNA sequencing of ectopic and eutopic endometrial tissues (3 ectopic *vs* 3 eutopic, **Supplementary Table S1**). As indicated in Principal Component Analysis (PCA), ectopic and eutopic endometrial tissues were significantly separated and the samples in each group were closely related (**Figure 2A**), suggesting that ectopic and eutopic endometrial tissues were transcriptionally different. Differential gene expression analysis showed that there were 1837 differentially expressed genes (DEGs) (**Figure 2B**) in the ectopic compared to eutopic samples, with 1079 genes upregulated and 758 downregulated. Next, we compared our RNA-seq data with another RNA-seq dataset comparing the ectopic with eutopic endometria in eight patients with ovarian endometriosis (GSE105764) (16). Venn plot showed that 70.4% upregulated and 60.95% downregulated DEGs in our RNA-seq data were overlapped with DEGs in GSE105764 dataset (**Figure 2C**), validating the reliability of our RNA-seq data. The top 10 upregulated and downregulated genes in ectopic endometrial tissues were shown in **Figure 2D**. The upregulated DEGs in ectopic samples were enriched in leukocyte migration, muscle system process, humoral immune in response (**Figure 2E**), and downregulated DEGs are enriched in functions such as nuclear division, chromosome segregation, and mitotic nuclear division (**Figure 2F**). In summary, ectopic and eutopic endometrial tissues were transcriptionally different from each other.

### Integrated analysis of DNA methylation and gene expression reveals candidate genes in endometriosis

**Figure 3:**
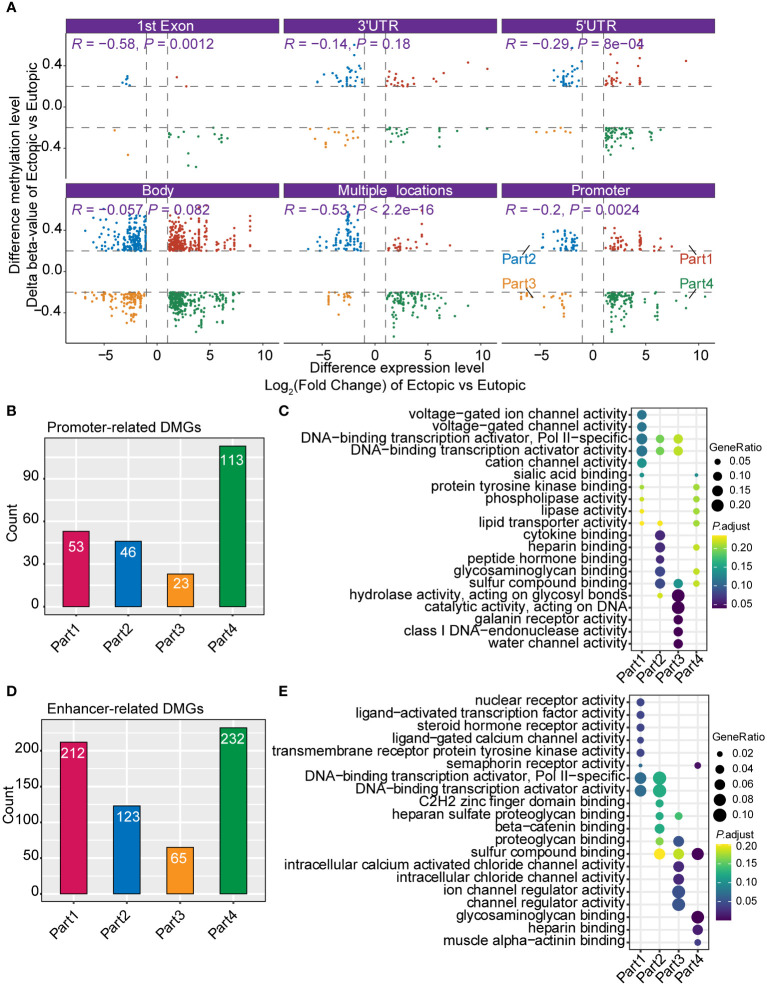
Integrated analysis of DNA methylation and gene expression profiles. **(A)** Correlation of the DNA methylation and gene expression grouped by different genomic positions (1st Exon, 3’UTR, 5’UTR, gene body, multiple locations, and promoter). **(B)** Bar plot shows the count of promoter-related differentially methylated genes (DMGs). **(C)** Gene Ontology (GO) of different groups of promoter-related DMGs (part1-part4 in **Figure 3A**). **(D)** Bar plot shows the count of enhancer-related DMGs. **(E)** GO analysis of different groups of enhancer-related DMGs.

DNA methylation was important in the gene expression regulation (8). To connect DNA methylation with gene expression in endometriosis, we further integrated our DNA methylation and RNA-seq data. First, we divided the DNA methylation states to six groups (1st exon, 3’UTR, 5’UTR, gene body, multiple locations, and promoter) according to the position of methylated loci. As expected, the level of DNA methylation (delta β-value) was negatively correlated with gene expression in most of the groups (1st exon, 5’UTR, multiple locations, and promoter) (**Figure 3A**). We further defined genes with differentially methylated loci in promoters as ‘promoter-related DMGs (differentially methylated genes)’ and genes with differentially methylated loci not in promoters as ‘enhancer-related DMGs’. According to the relation of level of methylation (delta β-value) and gene expression, genes were separated to four quadrants (part 1 to part 4, **Figure 3A**). Almost half of promoter-related DMGs (48.0%, 113/235) were in part 4 (**Figure 3B**). Promoter-related DMGs in part 4 were enriched in sialic acid binding and lipase activity (**Figure 3C**). Furthermore, promoter-related DMGs in part 2 were specifically enriched in channel activity. For enhancer-related DMGs, 70.25% were in part 1 and 4 (**Figure 3D**). DMGs in part 1 were enriched in transcriptional activity (nuclear receptor activity, ligand-activated transcription factor activity, and DNA-binding transcription activator), steroid hormone receptor activity and channel activity (**Figure 3E**). These results indicated that most DMGs in promoter and enhancer were negatively correlated with gene expression.

### Validation of candidate genes in endometriosis

**Figure 4:**
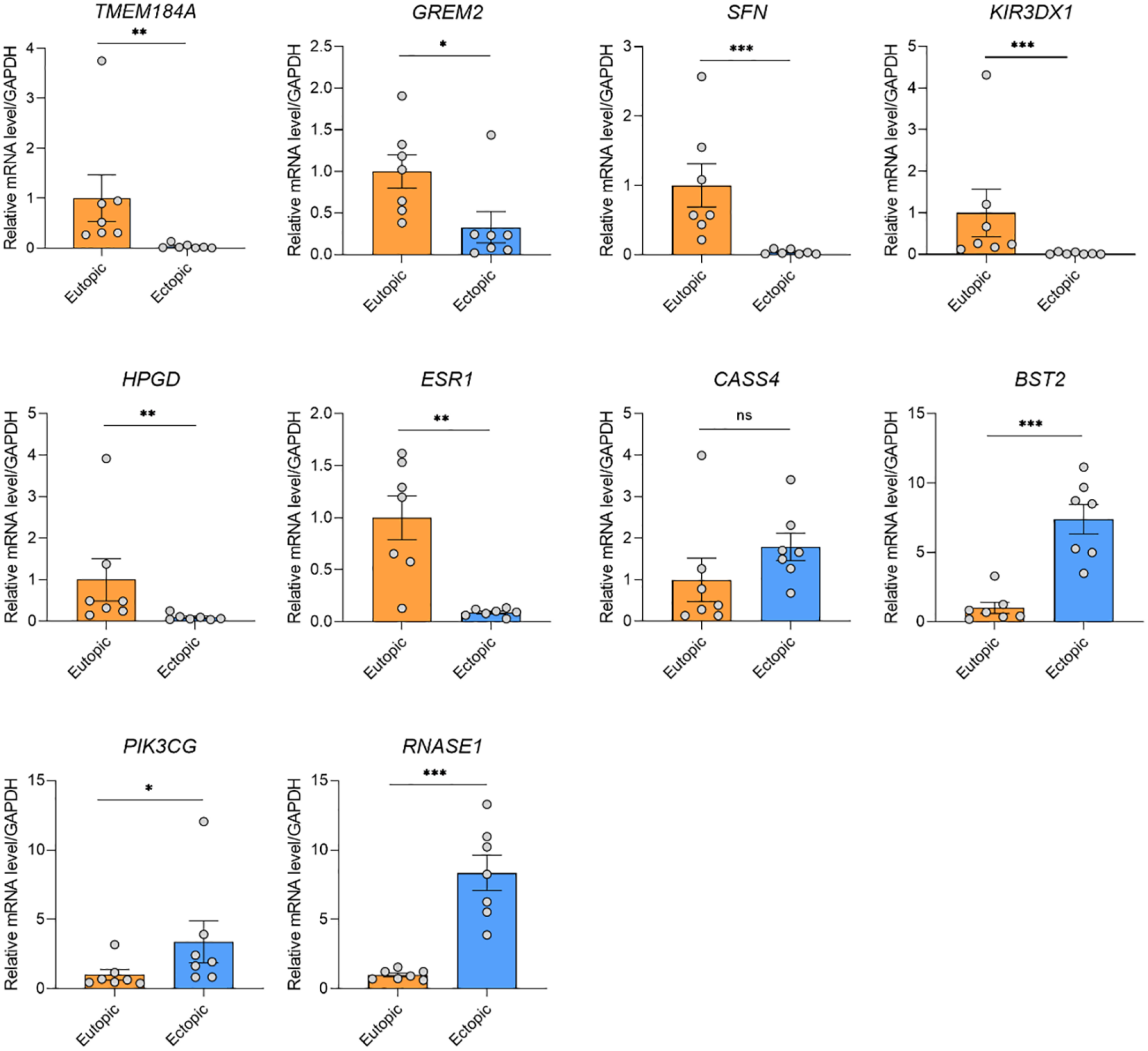
qRT-PCR validation of the gene expression of the candidate genes. *TMEM184A*, *GREM2*, *SFN*, *KIR3DX1*, *HPGD*, and *ESR1* were significantly downregulated in ectopic tissues, and *BST2*, *PIK3CG* and *RNASE1* were significantly upregulated in ectopic tissues. ***P < 0.001, **P < 0.01, *P < 0.05, ns, not significant.

To validate the candidate genes in endometriosis, we listed the top 15 DMGs (ranked by delta β-value) whose expression was upregulated (**Table 1**) and downregulated (**Table 2**). After filtering the past reported genes, 10 unreported DMGs (*TMEM184A*, *GREM2*, *SFN*, *KIR3DX1*, *HPGD*, *ESR1*, *CASS4*, *BST2*, *PIK3CG* and *RNASE1*) were kept. We further detected the expression of these 10 DMGs in ectopic and eutopic endometrial tissues (7 ectopic *vs* 7 eutopic, **Supplementary Table S1**). *TMEM184A*, *GREM2*, *SFN*, *KIR3DX1*, *HPGD*, and *ESR1* were significantly downregulated in ectopic tissues, and *BST2*, *PIK3CG* and *RNASE1* were significantly upregulated in ectopic tissues (**Figure 4**). In summary, we validated the top candidate genes in endometriosis which might be regulated by DNA methylation.

**Table 1:** Top candidate genes which were hypermethylated and downregulated in ectopic endometrial tissues.

Probe_ID	Gene	beta_case avg	beta_control avg	beta_change	RELATION_TO_CPG_ISLAND	log2FoldChange	padj
cg06738242	*TWIST2*	0.554053551	0.15610387	0.397949684	Island	-2.6571473	0.00015233
cg23210268	*GREM2*	0.699915101	0.30227628	0.397638816		-3.3286895	0.00018973
cg15188268	*ADAMTS19*	0.817201563	0.43219189	0.385009671	N_Shore	-3.6976664	6.26E-05
cg09386458	*QPCT*	0.886765946	0.51097633	0.375789613	N_Shore	-2.1138241	0.00406449
cg23037932	*KIR3DX1*	0.815017046	0.45292026	0.362096786		-4.7201206	0.00906221
cg22920700	*OSR2*	0.579597099	0.22700124	0.352595857	N_Shore	-1.7278745	0.02790383
cg01352551	*SLC24A3*	0.793752782	0.46820021	0.325552568	N_Shore	-1.5072888	0.03022538
cg23719157	*STRA6*	0.78738477	0.46561667	0.321768098		-2.4999297	0.01348782
cg13213527	*ZNF516*	0.775537145	0.45945426	0.316082885		-1.9614117	0.00040565
cg16549596	*SFN*	0.649653471	0.33389726	0.315756214	N_Shore	-2.9059607	0.00244178
cg04555941	*HPGD*	0.417078247	0.11941307	0.297665176	S_Shore	-2.5526777	0.00058597
cg00335591	*TMEM184A*	0.857047129	0.56488322	0.292163912		-1.8289676	0.04205992
cg25338972	*ESR1*	0.515218373	0.22405393	0.291164441		-2.5746547	0.00330811
cg13468624	*PSORS1C3*	0.694573016	0.41112169	0.283451322	N_Shore	-2.0015838	0.03245501
cg02992645	*OVGP1*	0.796760034	0.53732872	0.259431316		-2.9074884	0.00245663

**Table 2:** Top candidate genes which were hypomethylated and upregulated in ectopic endometrial tissues.

Probe_ID	Gene	beta_case_avg	beta_control_avg	beta_change	RELATION_TO_CPG_ISLAND	log2FoldChange	padj
cg24587601	*CASS4*	0.137575599	0.72258402	0.58500842		1.77916735	0.01566374
cg01329005	*BST2*	0.108425528	0.63957445	0.531148922		2.95333954	1.53E-05
cg13214190	*PIK3CG*	0.329104785	0.81439809	0.485293304	N_Shelf	1.75014039	0.01724619
cg13718960	*RNASE1*	0.327354776	0.79572139	0.468366613		2.68147978	1.05E-05
cg20986996	*TCF21*	0.211925743	0.67962279	0.467697044	N_Shore	6.09318374	5.74E-12
cg00123072	*THBS4*	0.453287548	0.88810567	0.434818118	N_Shore	2.15240829	0.00020269
cg26928972	*CSTA*	0.348577804	0.7816603	0.433082493		5.87099798	8.69E-13
cg15338159	*FHL2*	0.412303334	0.82354861	0.411245276		3.14284341	0.00012948
cg19876649	*MYOM1*	0.399121642	0.75975417	0.360632532		2.51276745	3.61E-05
cg13661129	*NR5A1*	0.469142795	0.82427195	0.355129158	S_Shelf	8.7981021	5.00E-19
cg24315815	*PLSCR4*	0.311694902	0.66008554	0.348390633	S_Shore	1.857896	0.01098051
cg04413226	*FMO1*	0.436500059	0.78104022	0.344540159		3.61678571	4.07E-09
cg01089639	*JAK3*	0.348527172	0.66969015	0.32116298	S_Shore	2.04877678	0.01319003
cg18525352	*NUAK1*	0.055519124	0.37199013	0.316471009	S_Shore	1.83605399	0.00401288
cg26683398	*LTC4S*	0.302200139	0.61640078	0.314200641	N_Shelf	3.06959132	4.57E-05

## Discussions

EMs, vividly known as ‘a kind of benign cancer’ and women’s pelvic ‘sandstorm’, has established itself as one of the most common diseases in gynecology by its high incidence and its early onset age, Symptoms like chronic pelvic pain, dysmenorrhea, and infertility caused by EMs have seriously threatened women’s health and reduced their life quality. EMs is one of the focuses of global reproductive health issues. According to the available literature reports, it is estimated that about 15% to 30% of women in the childbearing period would be affected by the disease, involving about 270 million people worldwide (17).

Although the theory of menstrual blood reflux proposed by Sampson J.J., who was the father of endometriosis in the world, has been accepted for a century and a half (5), the related factors and pathogenesis of EMs are still incomplete. Due to the wide range of lesions and diverse morphologies, malignant biological behaviors such as metastasis, invasion, and recurrence, and the possibility of malignant transformation, EMs is vividly called ‘benign cancer’ (1). More and more studies believe that its similar biological behavior to malignant tumors may be one of the important pathogenesis of EMs (18–21). The occurrence and development of malignant lesions are marked by the accumulation of tumor suppressor genes and oncogene genetic variations, especially by the abnormal methylation of tumor suppressor genes. Studies have confirmed that abnormal methylation of CpG islands regulated by non-methylated tumor suppressor genes is closely related to the occurrence and metastasis of human malignant tumors (22). A large number of studies have confirmed that EMs is a genetic disease caused by the interaction of multiple locus genes and environmental factors (23–26). HOXA10, hMLH1, PTEN promoter region 5’ CpG island methylation pattern is currently known to be related to the occurrence and development of EMs. HOXA10 gene can inhibit tumor cell proliferation, reduce invasion ability, and inhibit tumor cell metastasis. Recent studies abroad have found that the reduced expression of the HOXA10 gene in EMs is similar to malignant lesions. EMs animal model studies have further supported that methylation of the promoter region of the HOXA10 gene may be involved in the pathogenesis of EMs (27). PTEN is currently recognized as a tumor suppressor gene, and PTEN methylation is closely related to the occurrence and development of various tumors. Abnormalities in this gene have been demonstrated in many human cancers, especially in ovarian endometrioidal carcinomas, endometrial cancers, and gliomas (28). Salvesen et al. confirmed that PTEN gene methylation was prevalent in endometrial cancer, and tumor suppressor gene methylation was associated with advanced tumor metastasis in endometrial cancer and plays an important role in tumor progression (28). KF. Tam et al. (29) explored the relationship between DNA methylation and ovarian tumors, including PTEN genes. They concluded that the methylation rate of borderline ovarian tumors and ovarian cancer was significantly higher than that of benign ovarian tumors and normal ovarian tissue.

Our study identified a series of candidate genes and pathways in EMs. Of note, in KEGG pathway analysis, both hypermethylated and hypomethylated genes in ectopic were enriched in human papillomavirus (HPV) infection, suggesting a potential link between endometriosis and HPV infection. The prevalence of high-risk HPV was significantly higher in patients with EMs than in those without EMs (30). Besides, HPV-infected endometrial cells were reported to be spread through retrograde menstruation (31). The persistent HPV infection of endometriosis lesions was proposed to contribute to malignant progression. Since EMs is considered a common gynecologic problem with multifactorial origins, HPV infection is a highly possible pathophysiologic causes of EMs. We also validated the differential expression of top-ranked genes (*TMEM184A*, *GREM2*, *SFN*, *KIR3DX1*, *HPGD*, *ESR1*, *BST2*, *PIK3CG* and *RNASE1*) in EMs and normal tissues. *TMEM184A*, *GREM2*, *SFN*, *KIR3DX1*, *HPGD*, and *ESR1* were hypermethylated and significantly downregulated in ectopic endometrial tissues. Conversely, *BST2*, *PIK3CG* and *RNASE1* were hypomethylated and upregulated in ectopic endometrial tissues.

SFN is highly expressed in the esophagus-mucous membrane, skin, small salivary glands, and vagina (32–34), indicating its essential role in maintaining the homeostasis of a series of tissues and organs. The higher expression of SFN was significantly associated with a better prognosis of endometrial cancer (35). Accordingly, SFN was downregulated in ectopic endometrial tissues in our study, suggesting the loss of function of SFN might be involved in the pathophysiology of EMs. HPGD was reported to be involved in the pathophysiology of endometriosis (36). Decreased expression of HPGD is associated with abnormal prostaglandin metabolism in endometriosis (35, 37). Besides, the decreased expression of HPGD might be regulated by the miR-218-5p in endometrial adenocarcinoma tissue (35). ESR1 encodes estrogen receptors and ligand-activated transcription factors that can form homodimers or heterodimers with estrogen receptors. ERβ is widely expressed during endometrial hyperplasia in infertile women while decreasing or absent in women of normal childbearing age, suggesting that overexpression of ERβ may be related to infertility. Mutated ER in exon 8 with high activity *in vivo* may cause precocious puberty in girls (38). As reported, genetic variants in ESR1 were reported to change the susceptibility to endometriosis and might influence the fertility status in endometriosis patients (39). Moreover, ESR1 amplification might be one mechanism for ER over-expression in endometrial carcinoma (40). BST2 was one of the proteins that were found to be related to tumor metastasis (41). The anti-BST2 antibody had a potent antitumor effect against endometrial cancer both *in vitro* and *in vivo*, indicating that anti-BST2 antibody might be a potential therapeutic strategy for endometrial cancer. Interestingly, There are few studies of *TMEM184A*, *GREM2*, *KIR3DX1*, *BST2* and *RNASE1* in EMs, which needs more investigations to figure out their function in EMs in the future.

There are some limitations in this study. The low number of included patients might limit the statistical power of the findings. Further large-scale studies and investigations are needed to validate the findings. Besides, all the patients enrolled were Asian, which might introduce confounding when interpreting the results. With the increasing sequencing data of EMs being reported, meta-analyses of these sequencing data might give a more comprehensive view of the pathophysiology of EMs.

## Conclusion

In conclusion, we screened the differentially methylated and expressed genes through genome-wide DNA methylation and transcriptome sequencing of ectopic and eutopic endometrial tissues. Further integrated analysis identified a series of potential candidate genes in EMs. We also verified *TMEM184A*, *GREM2*, *SFN*, *KIR3DX1*, *HPGD*, *ESR1*, *BST2*, *PIK3CG* and *RNASE1* as key candidate genes in EMs.

## Data availability statement

The data presented in the study are deposited in the CNGB Sequence Archive (CNSA) of China National GeneBank DataBase (CNGBdb) repository, accession number CNP0003813.

## Ethics statement

The studies involving human participants were reviewed and approved by Ethics Committee of the Shanghai East Hospital of Tongji University. The patients/participants provided their written informed consent to participate in this study.

## Author contributions

FL and BL conceived and designed the project. LL and FL performed all operations. BL analyzed the data and drew the figures. LL and BL wrote the manuscript. XX, CG, BL and FL revised the manuscript. All authors contributed to the article and approved the submitted version.
