# Supplementary material for: Integrated analysis of genome-wide gene expression and DNA methylation profiles reveals candidate genes in ovary endometriosis
Source: Front Endocrinol (Lausanne). 2023 Mar 23;14:1093683. doi: 10.3389/fendo.2023.1093683 (PMC10076879; doi:10.3389/fendo.2023.1093683)
Supplement: Supplementary file 1 [file Table_1.docx]

**Supplemental Tables**

**Supplemental Table S1. The clinical information of patients enrolled in this study.**

| **No.** | **Age** | **Gestation** | **Parity** | **Allergy** | **Chronic or gyn disease** | **Surgery**  **history** | **Applications** |
| --- | --- | --- | --- | --- | --- | --- | --- |
| 1 | 34 | 1 | 1 | - | - | Cesarean section | DNA methylation array |
| 2 | 32 | 0 | 0 | - | - | - | DNA methylation array |
| 3 | 31 | 3 | 1 | - | - | Cesarean section | DNA methylation array |
| 4 | 42 | 2 | 1 | - | - | - | DNA methylation array |
| 5 | 36 | 5 | 2 | - | - | - | DNA methylation array |
| 6 | 29 | 0 | 0 | - | - | - | DNA methylation array |
| 7 | 39 | 0 | 0 | - | - | - | qRT-PCR, RNA-seq |
| 8 | 30 | 0 | 0 | - | - | - | qRT-PCR, RNA-seq |
| 9 | 31 | 0 | 0 | - | - | - | qRT-PCR, RNA-seq |
| 10 | 31 | 1 | 0 | - | - | - | qRT-PCR |
| 11 | 39 | 1 | 1 | - | - | Cesarean section | qRT-PCR |
| 12 | 35 | 1 | 1 | - | - | Cesarean section | qRT-PCR |
| 13 | 27 | 0 | 0 | - | - | - | qRT-PCR |

(-): no history of allergies, chronic diseases, other gynecological diseases, or surgery.

**Supplemental Table S2. Primers used in the qPCR validations.**

| Genes | Primers |
| --- | --- |
| *TMEM184A*-F | TCGTGGAAAGCCCATCAAGT |
| *TMEM184A*-R | AGGAACCCGATGGAGTAGGT |
| *GREM2*-F | CGAGCGCAAGTACCTCAAGA |
| *GREM2*-R | GTAGCAGAAGCGGTTGAGGA |
| *SFN*-F | ACTACGAGATCGCCAACAGC |
| *SFN*-R | CAGTGTCAGGTTGTCTCGCA |
| *KIR3DX1*-F | AAAGAGGGGCACATACAGCA |
| *KIR3DX1*-R | GACAGCCTCGACGTAATGGA |
| *HPGD*-F | ATGCACGTGAACGGCAAAGT |
| *HPGD*-R | ATCCAGGGCAGCTTTACACT |
| *ESR1*-F | CCAGATGGTCAGTGCCTTGT |
| *ESR1*-R | CATGTGAACCAGCTCCCTGT |
| *CASS4*-F | CCCACCAGTGCCAGAATCAT |
| *CASS4*-R | GCACGTCATACACCTGGGAA |
| *BST2*-F | ATGGAAGACGGGGATAAGCG |
| *BST2*-R | GGAGATGGGTGACATTGCGA |
| *PIK3CG*-F | AGAAGACAAGCCCACACTTCC |
| *PIK3CG*-R | TTGTGTGATGACGAAGGGCTA |
| *RNASE1*-F | AACTGTGCATACCGGACCAG |
| *RNASE1*-R | AGCATCAAAGTGGACTGGCA |
| *GAPDH*-F | GACTCATGACCACGTCCATGC |
| *GAPDH*-R | AGAGGCAGGGATGATGTTCTG |
